# Miscellaneous, &c.

**Published:** 1894-12-22

**Authors:** 


### MISCELLANEOUS.—SPECIAL.

**Dental Hospital of London**, Leicester Square.—It is intended to remove this useful special hospital to a new site as soon as the money required is forthcoming, the sanitation and accommodation of the present building being inadequate; the amount now required for this is £14,000. Dentistry is a branch of medicine that is justified in having a special hospital, and the committee can assure the benevolent public that the Dental Hospital of London is in every respect worthy of support. Help by an increase in annual subscriptions is also earnestly solicited. Its bankers are Messrs. Barclay, Ransom, and Co., and the Secretary is Mr. J. Francis Pink.

**Hospital for Diseases of the Throat**, Golden Square, Regent Street, W.—The committee appeal for donations and subscriptions towards the maintenance of the children's ward, and they earnestly hope that some portion of the charitable gifts of the season will be devoted to this object. The special ward recently opened for children is kept constantly full, and a very large number of children are awaiting their turn for admission. Secretary, W. Holt; Matron, Miss M. Bywater.

**Lock Hospital and Home**, Harrow Road, W.—Rescue work, as well as the cure of the patients who present themselves for admission, is the aim of this institution, and the good work has only to be known to meet with the support it deserves. Considerably more than one-fourth of those that pass through the hospital are reclaimed from their evil lives, which is a great result. A debt of £2,500 has to be cleared off, and £1,000 is required in fresh annual subscriptions to meet the regular requirements of the institution. Secretary, Mr. A. W. Cruikshank; Matron of hospital, Mrs. Kydd; Matron of home, Miss J. McNiven.

**Royal London Ophthalmic Hospital**, Moorfields, E.C.—An urgent appeal is made by the Board of Management for funds in support of this institution. The expenditure amounts annually to over £6,000, whereas the certain income is about £2,000. The patients numbered in 1893 a total of 26,927; of these 1,972 were in-patients, and the total attendances of the out-patients was 116,775. All patients are admitted free, and without letters of recommendation. Bankers: Williams, Deacon, and Co., 20, Birchin Lane, E.C.; Secretary, Mr. Robert J. Newstead; Matron, Miss Nichol.

**National Dental**, Great Portland Street.—The fine new building, at the corner of Great Portland Street and Devonshire Street, which takes the place of the old house, 149, Great Portland Street, is now in full working order. The Duke of York has become the new President, the Earl of Strafford Vice-President, and Mr. Charles Hoare Treasurer. Secretary, E. Almack.

